# Preparation, Physicochemical Properties, Biological Activity of a Multifunctional Composite Film Based on Zein/Citric Acid Loaded with Grape Seed Extract and Its Application in Solid Lipid Packaging

**DOI:** 10.3390/foods14101698

**Published:** 2025-05-11

**Authors:** Ning Wang, Jiaxin Wei, Cuntang Wang, Jian Ren

**Affiliations:** 1 College of Food and Bioengineering, Qiqihar University, Qiqihar 161006, China; 2 Engineering Research Center of Plant Food Processing Technology, Ministry of Education, Qiqihar 161006, China

**Keywords:** zein, grape seed ethanol extract, bio-based composite film, antioxidant property, antibacterial property, soil degradability, food packaging

## Abstract

Development of bio-based active packaging systems for lipid stabilization presents critical importance in preserving lipid integrity and ensuring food safety. Zein/citric acid (Z/CA) composite films containing grape seed ethanol extract (GSEE) (0–8% *w/w*) were prepared by the solvent casting method. The structural, functional, and environmental properties of the films, including physical and chemical properties, mechanical properties, antioxidant capacity, antibacterial activity, oxidation inhibition effect, and biodegradability, were comprehensively characterized and evaluated. Progressive GSEE enrichment significantly enhanced film thickness (*p* < 0.05), hydrophobicity, and total phenolic content, while increasing water vapor permeability by 61.29%. Antioxidant capacity demonstrated radical scavenging enhancements of 83.75% (DPPH) and 89.33% (ABTS) at maximal GSEE loading compared to control films. Mechanical parameters exhibited inverse proportionality to GSEE concentration, with tensile strength and elongation at break decreasing by 28.13% and 59.43%, respectively. SEM microstructural analysis revealed concentration-dependent increases in surface asperity and cross-sectional phase heterogeneity. Antimicrobial assays demonstrated selective bacteriostatic effects against Gram-negative pathogens. Notably, the composite film containing 6 wt% GSEE had a remarkable restraining effect on the oxidation of lard. The soil degradation experiment has confirmed that the Z/CA/GSEE composite film can achieve obvious degradation within 28 days. The above results indicate that the Z/CA/GSEE composite material emerges as a promising candidate for sustainable active food packaging applications.

## 1. Introduction

In food processing, the appropriate selection of food packaging materials is crucial for maintaining the quality of food and extending its shelf life. Traditional petroleum-based plastic films have been widely used as packaging materials because of their low density, low cost, and excellent molding properties [1]. However, because of their non-biodegradable nature and the potential risk of migrating harmful components to food during the storage stage, the environment and consumers’ health have been seriously threatened [2]. Consequently, bioactive and biodegradable green packaging materials are now a research focus and major development direction in food packaging [3]. Currently, biodegradable materials, including polysaccharides (such as starch, cellulose, and chitosan), proteins (like soy protein, zein, and whey protein), and lipids (for example, paraffin and shellac resin), have been utilized in the preparation of biodegradable films [4].

Zein is produced as a by-product of the corn starch processing industry. This protein is rich in hydrophobic amino acids, especially proline and glutamine. Still, it lacks essential amino acids such as lysine and tryptophan, so its nutritional value is relatively limited [5]. Zein has good film-forming ability and can be made into continuous, transparent films with specific mechanical strength. Its molecules are held together by a multitude of hydrogen bonds and hydrophobic interactions, which contribute to the structural stability of the film [6]. However, pure zein films have several limitations. For example, they exhibit poor flexibility, suboptimal barrier properties, and reduced mechanical strength in humid environments, which limits their widespread application in food packaging [7]. To improve the mechanical properties of zein films, researchers have tried to modify them using physical, chemical, or biological methods [8]. Citric acid, a common organic acid, is a cross-linker in zein film systems. Through its interaction with zein molecules, the arrangement and aggregation state of molecular chains can be changed, thereby enhancing the flexibility and ductility of the film and improving its mechanical and barrier properties [9]. However, simultaneously achieving antioxidant and antibacterial functions remains the major challenge for functional packaging films.

The wine industry is one of the significant agricultural product processing sectors globally, generating a substantial amount of solid by-products annually during the production process. Statistics reveal that these related processing activities yield approximately 14.5 million tons of waste materials each year, primarily consisting of grape pomace (including grape skins, stems, and seeds, among others), which constitutes about 20–30% of the raw grapes [10]. Within these waste materials, the proportion of grape seeds fluctuates based on the grape variety and processing technique, typically ranging from 5% to 10% [11]. In the food industry, grape seeds are usually used as compost or animal feed. However, the polyphenolic substances with various biological activities contained in them have not been exploited yet. According to relevant reports, grape-seed extracts are rich in polyphenolic substances such as catechin, epicatechin, gallic acid, and proanthocyanidins, and these substances have been proven to possess good antibacterial, antioxidant, anti-inflammatory, and other biological activities [12,13]. When the GSEE is added to the film, it can effectively reduce the growth rate of bacteria and the oxidation rate of fat, and maintain the color stability of the product under low-temperature storage conditions [14].

Currently, researchers are incorporating extracts from various biological wastes into bio-based films. For example, extracts from onion skins [15], lemon peels [16], grape pomace [17], watermelon rinds [18], water chestnut peels [19], and walnut shells [20] have been used to blend with film-forming matrices to prepare active films.

Therefore, the grape-seed extract is added to the zein-citric acid composite film. This measure can not only effectively utilize this agricultural waste resource to achieve the goal of resource reuse, but also endow the composite film with excellent antioxidant and antibacterial properties. Currently, while polyphenolic biofilms have been extensively researched, this study diverges from previous work that concentrated solely on the direct effects of grape seed ethanol extract. The zein/citric acid matrix serves as a synergistic platform, enhancing the stability and controlled-release properties of grape seed ethanol extract and addressing the issue of rapid degradation of polyphenolic substances [21].

During the storage and transportation stages, solid fats are highly susceptible to the effects of metal ions, oxygen, and microorganisms, which leads to the deterioration of their quality and flavor [22]. Current research mainly focuses on the antioxidant packaging of liquid fats, but there is insufficient attention paid to the active packaging materials for solid fats. On this basis, a ternary composite film system of grape seed extract-zein-citric acid was innovatively constructed. The effects of different concentrations of grape seed extract on the mechanical properties, barrier properties, and antioxidant and antibacterial properties of the zein/citric acid (Z/CA) composite films were systematically analyzed. Through the analysis of the microstructure of the film, the relationship between the structure and properties of the film was revealed in detail. In addition, the inhibitory effect of the composite film on the peroxide value during the storage of solid fats was also analyzed, thus providing theoretical support for the development of environmentally friendly and functional packaging for lipid-based food products.

## 2. Materials and Methods

### 2.1. Materials

Grape seeds and lard were from Jiefangmen Market in Qiqihar, China (Qiqihar, China). Zein (Sinopharm Chemical Reagent Co., Ltd., Shanghai, China) was from Shanghai Aladdin Biochemical Technology Co., Ltd. The yeast extract was sourced from Guangdong Huankai Microbial Technology Co., Ltd. (Guangzhou, Guangdong, China). DPPH and ABTS were from Sigma-Aldrich Chemical Company in the United States (Sigma-Aldrich Chemical Company, St. Louis, MO, USA). Acquisition of additional chemical reagents was conducted through Tianjin Kaitong Chemical Reagent Co., Ltd. (Huankai Microbial Technology Co., Ltd., Guangzhou, Guangdong, China).

### 2.2. Preparation of GSEE

Anthocyanins extraction from grape seeds was modified according to the method of Wang et al. [23]. GSEE was stored in a −20 °C for use.

### 2.3. Preparation of Z/CA/GSEE Composite Film

The Z/CA/GSEE films were prepared by the solution casting method according to the method of Wang et al. [23]. The films were named Z/CA/GSEE-0%, Z/CA/GSEE-2%, Z/CA/GSEE-4%, Z/CA/GSEE-6%, and Z/CA/GSEE-8% according to the amount of GSEE added.

### 2.4. Characterization

#### 2.4.1. Fourier Transform Infrared Spectrum (FTIR) Determination

Fourier transform infrared spectroscopy of Z/CA/GSEE films was determined according to the method of Qin et al. [24].

#### 2.4.2. X-Ray Diffraction Analysis (XRD)

The parameters of the instrument were set as 45 kV and 200 mA. The diffraction angle was set such that 2θ ranged from 5° to 60°. The scanning speed was set at 4° per minute [24].

#### 2.4.3. Scanning Electron Microscopy (SEM)

The microscopic structure of the Z/CA/GSEE films was analyzed using a scanning electron microscope ((S-4300, Hitachi, Tokyo, Japan) according to the method described by Wang et al. [25].

#### 2.4.4. Determination of Thickness

The thickness of the Z/CA/GSEE film was measured following the approach proposed by Chen et al. [26].

#### 2.4.5. Optical Properties

The light values of a*, b* and L* for the film samples were measured by referring to the method of Gao et al. [27].

The opacity was measured according to the method of Sukhija et al. [28].

#### 2.4.6. Determination of Mechanical Properties

The mechanical characteristics of Z/CA/GSEE coatings were assessed using the protocol outlined by Huang et al. [29].

#### 2.4.7. Determination of Water Vapor Transmittance (WVP)

The WVP determination method of Z/CA/GSEE film was adopted by Gao et al. [27].

#### 2.4.8. Determination of the Water Contact Angle (WCA)

The WCA of the film samples were measured by referring to the method of Lei et al. [30].

#### 2.4.9. Total Phenolic Content and Radical Scavenging Properties of Composite Films

The Z/CA/GSEE films’ overall phenolic concentration was assessed utilizing the protocol described by Xie et al. [31], and the results were expressed as GAE mg/dw/g (GAE: gallic acid). The DPPH and ABTS radical scavenging capacities of the Z/CA/GSEE films were measured using the method of Gao et al. [27].

#### 2.4.10. Antibacterial Activity of Composite Films

The antimicrobial activity of the composite membranes was performed using the method described by Meng et al. [32] with some modifications.

#### 2.4.11. Determination of the Degradability of the Composite Membrane Soil

The soil degradability of the composite film was determined according to the method of Su et al. [33].

#### 2.4.12. Determination of Peroxide Value (POV) of Composite Film Packaging Lard

The POV are determined according to the method of Gao et al. [27]. Each sample is measured three times, and the results are averaged.

#### 2.4.13. Data Processing

Experimental results were reported as mean values ± standard deviations. To analyze the significance of data differences, Duncan’s multiple range test in SPSS 26 software (SPSS Inc., Chicago, IL, USA) was employed. Origin 2022 software (Microsoft, WSU, USA) was utilized to plot the experimental data.

## 3. Results and Discussion

### 3.1. FTIR Analysis of the Thin Film

**Figure 1:**
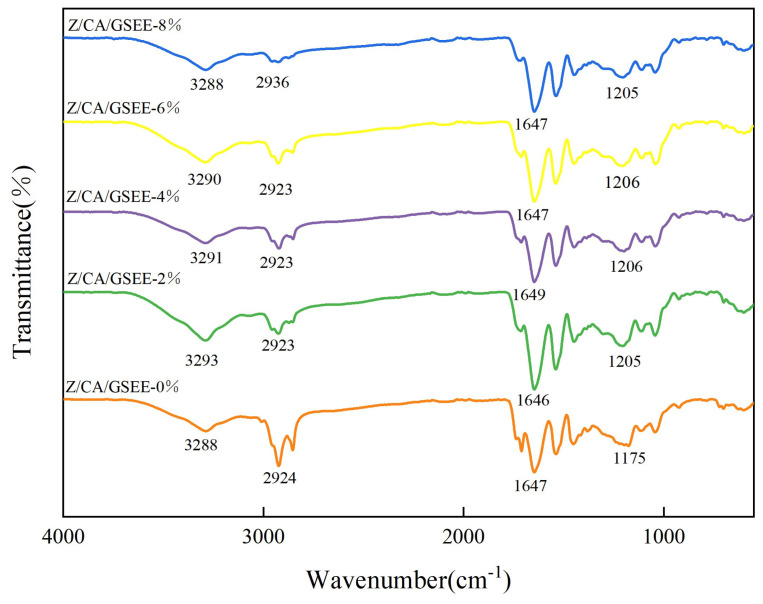
FTIR of Z/CA/GSEE−0%, Z/CA/GSEE−2%, Z/CA/GSEE−4%, Z/CA/GSEE−6% and Z/CA/GSEE−8% films.

Through infrared spectroscopy analysis, the interactions and changes in chemical compositions within the composite film were studied [34]. It can be seen from Figure 1, the characteristic peak in the spectrum of the Z/CA/EEOS-0% film at 3288 cm^−1^ corresponds to the O-H stretching vibration. The peak at 2924 cm^−1^ can be attributed to the C-H stretching vibration [35].

The C=O stretching vibration corresponds to an absorption peak located at 1647 cm^−1^, and the peak at 1175 cm^−1^ indicates the symmetrical C-O stretching vibration. Compared with the Z/CA/GSEE-0% film, the film with the addition of GSEE did not show additional peaks, indicating that the addition of GSEE will not change the chemical structure of the film. As the concentration of GSEE increases, the O-H stretching vibration shifts from 3290 cm^−1^ to 3288 cm^−1^. This shift is likely due to the formation of hydrogen bonds between the hydroxyl groups of GSEE and the amino groups of zein, thereby enhancing the intermolecular interactions within the film matrix. Tao et al. [36] reported that when the extract of Chinese gall was incorporated into the chitosan film, a similar shift in the O-H stretching vibration also occurred, indicating the enhancement of hydrogen bonds, thereby improving the stability of the film.

### 3.2. X-Ray Diffraction (XRD) Analysis of the Film

**Figure 2:**
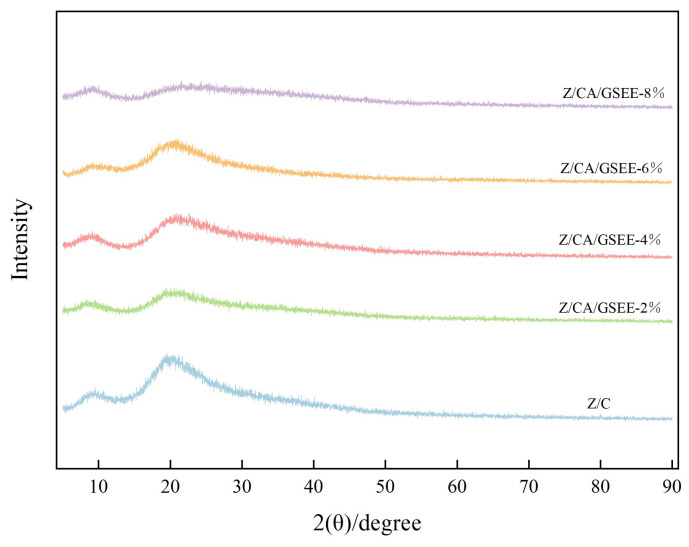
XRD patterns of Z/CA/GSEE−0%, Z/CA/GSEE−2%, Z/CA/GSEE−4%, Z/CA/GSEE−6% and Z/CA/GSEE−8% films.

X-ray diffraction (XRD) is a benchmark method for characterizing amorphous structures of materials [3]. The XRD patterns of the Z/CA/GSEE composite membranes with different concentrations of GSEE are shown in Figure 2. The diffraction peaks at approximately 10° and 20° (2θ) correspond to the α-helix and β-sheet structures of zein, respectively. As the GSEE concentration increased from 0 wt% to 8 wt%, a reduction in the intensity of the diffraction peaks, which implied a reduction in crystallinity. This is because GSEE disrupts the secondary structure of zein via hydrogen bonding and hydrophobic interactions and competes with citric acid for cross-linking sites. Ultimately, it diminishes the crystallinity of the composite film, causing the film structure to shift towards an amorphous state [37]. The reduction in crystallinity due to the rise in GSEE concentration is in line with the research findings of Zhang et al. [38]. The addition of black plum extract also lessens the intensity of the film’s diffraction peaks as the extract rich in anthocyanins disrupts the ordered packing of the film. However, incorporating purple corn extract can boost the intensity of the film’s diffraction peaks, and this might be due to the creation of a more ordered film structure [24]. Consequently, the crystallinity of the anthocyanin-rich film is notably influenced by the source of anthocyanins.

### 3.3. Analysis of the Microstructure

**Figure 3:**
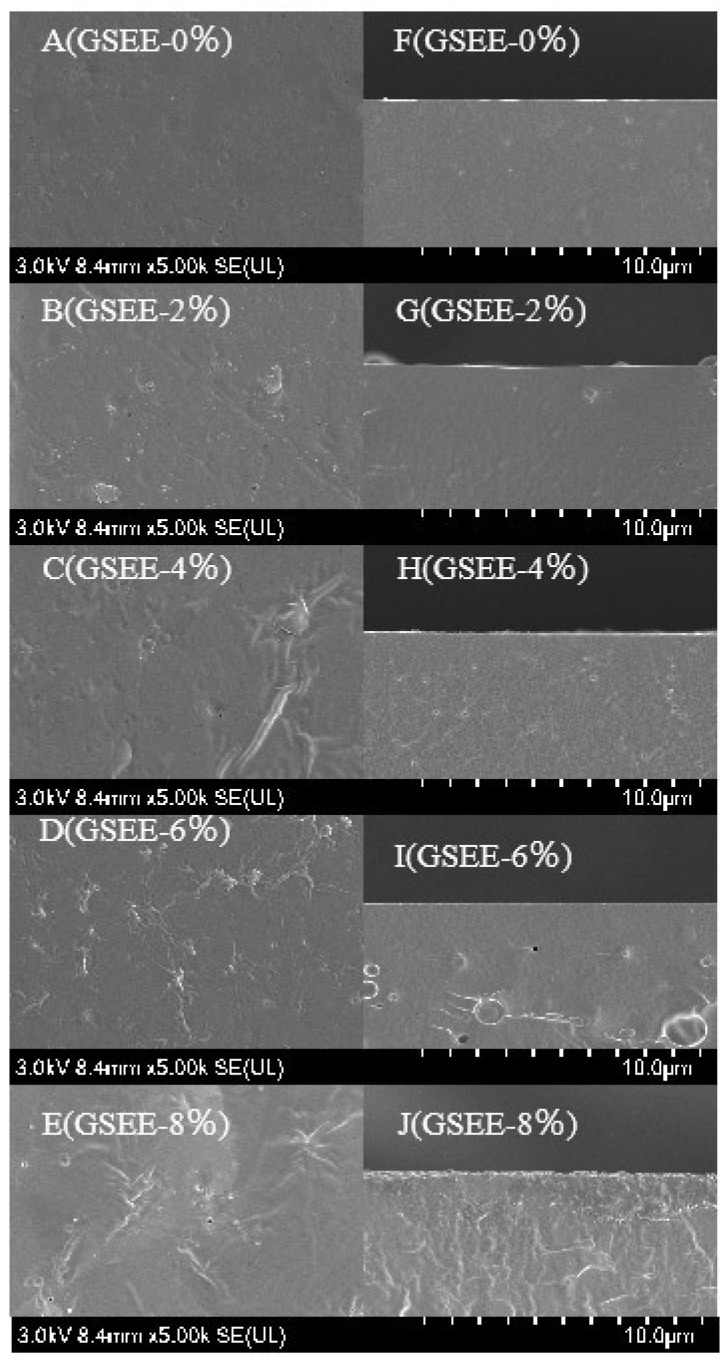
SEM images presenting the surface (**A**–**E**) and cross-section (**F**–**J**) of the film.

The microstructure of the film is determined by the interactions among its constituent materials, and these interactions will further affect various properties of the film, such as thickness, optical properties, mechanical strength, barrier properties, and antibacterial properties [16]. The surface morphology of zein-based films containing different contents of GSEE is shown in Figure 3. The surface of the composite film without GSEE (Figure 3A) exhibits smooth and flat characteristics, without noticeable particles or defects, which indicates that zein/citric acid and GSEE have good compatibility, forming a uniform matrix structure. Its cross-section (Figure 3F) shows relatively dense properties without noticeable voids or delamination. When the GSEE concentration rises to 2 wt%, minuscule irregular protrusions emerge on the film surface (Figure 3B), potentially stemming from the interaction between the GSEE components and the matrix. The cross-section, as shown in Figure 3G, maintains a relatively consistent appearance. Nevertheless, some minute structural alterations are discernible. This indicates that the incorporation of GSEE has already begun to exert an influence on the film’s internal structure. When the GSEE concentration reaches 4 wt%, the irregularity of the film surface (Figure 3C) becomes more apparent, and more dispersed small particles appear, likely due to the aggregation of certain substances in GSEE. The cross-section (Figure 3H) shows some uneven layers, indicating that the further increase in the GSEE concentration has led to the reorganization of the internal structure of the film. When the GSEE concentration is 6 wt%, the particles on the film surface (Figure 3D) become larger and more unevenly distributed, and the surface roughness increases significantly. Apparent voids and discontinuous areas can be seen in the cross-section (Figure 3I), which may affect the film’s performance. When the GSEE concentration reaches 8 wt%, the film surface presents a highly irregular morphology, with many larger particles and uneven areas. The cross-section (Figure 3J) shows severe structural disorder, with large voids and delamination. The structural changes in the film observed in this study are somewhat related to the previous research results. For instance, in the research conducted by Wang et al. [18], comparable surface and internal structural modifications were noted when watermelon peel extract was incorporated into the film matrix composed of chitosan (CS) and guar gum (GG). They discovered that with the rise in the extract’s concentration, the film’s surface roughness grew. This was due to the aggregation phenomenon stemming from the interaction between the bioactive components within the extract and the polymer matrix. Likewise, in the work of Nor Adilah et al. [39], mango peel extract (MPE) was incorporated into fish gelatin film for active food packaging applications. As the concentration of mango peel extract in the gelatin film was raised from 1% (*w/w*) to 5% (*w/w*), a comparable granular manifestation was also detected.

### 3.4. The Thickness, Color, and Opacity of the Composite Film

The thickness of the composite film serves as a vital parameter in the evaluation of its performance as a food packaging material [40]. The thickness of the film has a significant impact on the light transmittance, WVP, and mechanical strength [41]. As depicted in Table 1, within the scope of this investigation, the thickness, color parameters, and opacity of the Z/CA/GSEE composite film underwent analysis. With the upward trend of the GSEE concentration, there was a significant increase in the thickness of the film. When the GSEE concentration reached 8 wt%, the thickness increased by 44.76%. Firdaus et al. [16] investigated a packaging film made from lemon peel pectin and chitosan, into which they incorporated a bioactive extract from neem leaves. They found that, when the concentration of the neem leaf extract was raised to 2%, the thickness of the film increased by 53.57%. Such a tendency results from the rise in the solid content in the film matrix and the interference with the film’s ordered structure caused by the extract droplets. Conversely, the research carried out by Kahya et al. [42] indicated that, upon integrating sage and rosemary extracts into the chitosan film at a volume ratio of 1:4, the film thickness dropped from 0.056 mm to 0.026 mm.

Conversely, the visual characteristics of food packaging are of vital significance in shaping consumers’ perception. Plant extracts rich in polyphenols may interact with biopolymers, thus leading to color changes [43]. As the concentration of GSEE rose, the color parameters (L*, a*, and, b*) exhibited notable alterations. When the GSEE concentration increased from 0 wt% to 8 wt%, the L* value decreased by 20.87%, suggesting that the color of the film deepened. The a* value was significantly increased by 76.19%, while the b* value representing the degree of yellowness decreased by 24.43%. These changes indicate that the incorporation of GSEE made the film exhibit a reddish tone, which is mainly caused by the added polyphenolic extract. Moreover, to fulfill consumers’ expectations of visually assessing the contents of packaged food, the packaging film must possess a certain level of transparency. However, incorporating grape seed extract into the zein-based film will reduce its transparency, which is directly related to the concentration of GSEE. As the GSEE concentration rose, the opacity of the film increased steadily. Compared with the composite film without GSEE, when the GSEE concentration was 8 wt%, the opacity increased by 43.26%. This finding aligns with the research outcomes of S.G. et al. [44]. When they added plant extracts to the sodium carboxymethyl cellulose (CMC) and gelatin (GA) matrix, they found that, with the addition of basil leaf (RL) extract and mint (MP) extract, the transparency of the CMC film decreased by 38.91% and 30.95%, respectively. Although opacity helps protect light-sensitive foods from UV degradation, excessive opacity may have a negative impact on the appeal to consumers.

### 3.5. Analysis of Mechanical Properties

**Figure 4:**
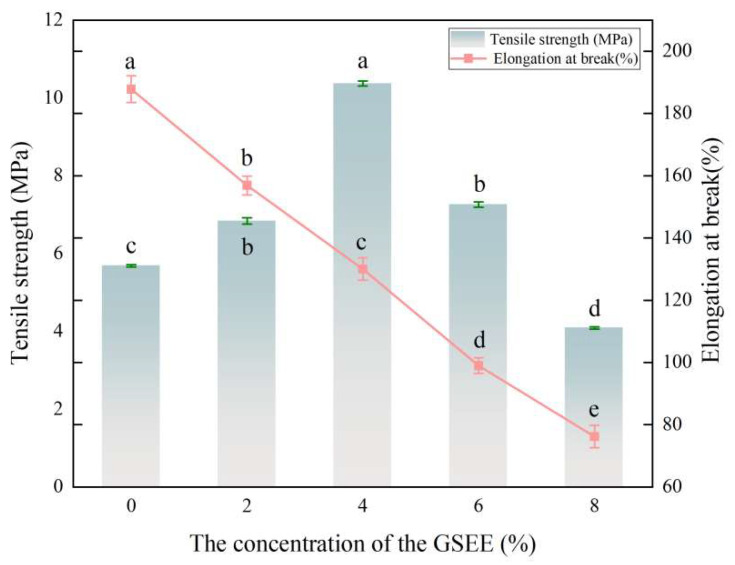
The mechanical properties of the Z/CA/GSEE−0%, Z/CA/GSEE−2%, Z/CA/GSEE−4%, Z/CA/GSEE−6% and Z/CA/GSEE−8% films. ^a–e^ Values are given as means + SD. Different letters in the figure indicate significant difference (*p* < 0.05).

The mechanical properties of the film are typically quantified by the tensile strength (TS) and the elongation at break (EAB). These two properties are considered the crucial factors for gauging the strength and flexibility of the material [45]. As shown in Figure 4, as the quantity of GSEE increased, the EAB of the zein-based film decreased significantly. Conversely, the TS exhibited a pattern of initially rising and subsequently declining (*p* < 0.05). Specifically, when the addition amount of GSEE increased from 0 wt% to 8 wt%, the elongation value at break was reduced by 59.43%. Regarding the TS, when the extract concentration increased to 4 wt%, the film’s TS reached a maximum of 10.37 MPa. However, when the extract concentration was further raised to 8 wt%, the TS declined, dropping by 28.13% compared to the film without the extract. This may be because GSEE increases the active sites available for cross-linking in zein molecules, thereby improving the cross-linking efficiency of citric acid and enhancing the tensile strength. Subsequently, an excessive amount of GSEE may occupy the binding sites of citric acid, leading to the fragmentation of the network structure. This, in turn, causes a decrease in the overall strength of the material and simultaneously reduces the elongation at break [46]. Silva et al. [3] found that, when the extract of Schinus terebinthifolia leaves was added to the yam starch film matrix, with the continuous increase in the extract concentration, the mechanical properties of the film decreased accordingly. This occurs due to the absence of intermolecular interactions between the extract of Schinus terebinthifolia leaves and yam starch. As a result, the flexibility of the film is impeded. Meng et al. [47] also found similar results in the study of chitosan–starch films incorporated with peanut shell extract. They held the view that the addition of plant extracts abundant in polyphenols led to this reduction in flexibility.

### 3.6. Water Vapor Permeability (WVP)

**Figure 5:**
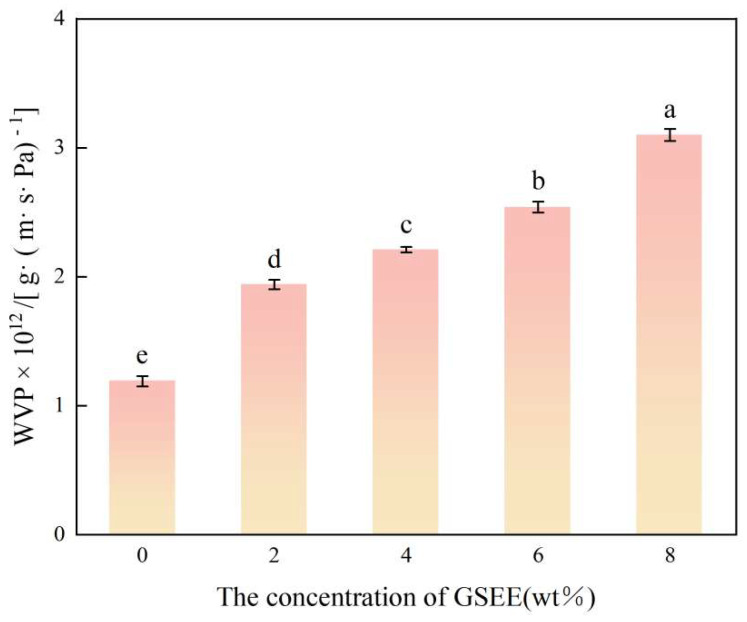
The WVP of Z/CA/GSEE−0%, Z/CA/GSEE−2%, Z/CA/GSEE−4%, Z/CA/GSEE−6% and Z/CA/GSEE−8% composite films. ^a–e^ Values are given as means + SD. Different letters in the figure indicate significant difference (*p* < 0.05).

WVP is regarded as one of the critical indicators for measuring the moisture-proof performance of packaging films, and it is directly associated with the quality and durability of packaged food [48]. The correlation between the GSEE concentration and the WVP value is shown in Figure 5. The study indicates that, as the GSEE concentration increases from 0 wt% to 8 wt%, the WVP value shows a distinct upward trend. When the GSEE concentration is 0 wt%, the WVP value is approximately 1.2 × 10 g/(m·s·Pa). When the GSEE concentration is increased to 8 wt%, the WVP value is increased by 61.29%. This may be due to the fact that the addition of GSEE disrupts the ordered structure of zein, forming a looser amorphous network. Such structural changes may generate more discontinuous micro-pore channels, providing pathways for the diffusion of water molecules, thus facilitating the penetration of water vapor [49]. This is consistent with the results of X-ray diffraction (XRD). Similar research findings indicate that Wang et al. [23], while investigating the active film made from corn starch/carrageenan and grape seed extract, discovered that as the concentration of GSEE rose from 0% to 5%, the WVP went up by 31.65%. Song et al. [50] found in their study on the preparation of composite active packaging films using tapioca starch/pectin (TSP) and broccoli leaf polyphenols (BLP) as raw materials that as the amount of BLP added increased, the WVP gradually decreased. However, when the addition amount of BLP reached 5%, the WVP increased compared with that when the addition amount was 3%. This increase may be due to the fact that BLP increased the thickness and density of the TSP + BLP composite film, thus hindering the penetration of water molecules.

### 3.7. Antioxidant Activity

**Figure 6:**
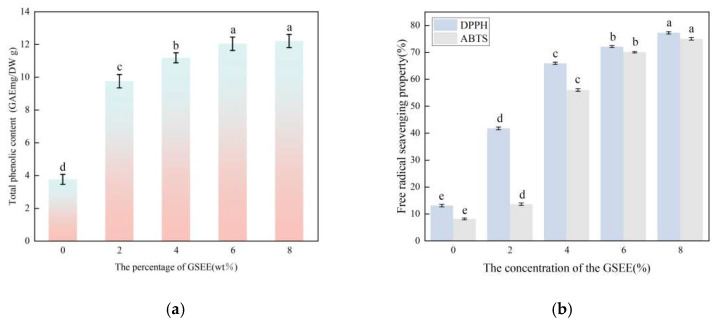
TPC (**a**) and DPPH and ABTS free radical scavenging activities (**b**) of Z/CA/GSEE−0%, Z/CA/GSEE−2%, Z/CA/GSEE−4%, Z/CA/GSEE−6% and Z/CA/GSEE−8% films. ^a–e^ Values are given as means + SD. Different letters in the figure indicate significant difference (*p* < 0.05).

Total phenolic content (TPC) is employed to assess the quantity of phenolic compounds within the sample. Specifically, this activity serves to identify the samples abundant in polyphenols and flavonoids, and these compounds are capable of functioning as free radical scavengers and oxidative stress alleviators [51]. Figure 6a depicts the alterations in the TPC within the composite films featuring varying amounts of GSEE addition. As the GSEE addition amount increases from 0 wt% to 8 wt%, the TPC of the composite film shows a distinct upward trend (*p* < 0.05). When the GSEE addition amount is 0 wt%, the TPC is approximately 4 GAEmg/DW g, and when the addition amount reaches 8 wt%, the TPC is increased by 69.29%. This indicates that the addition of GSEE can significantly increase the TPC of the composite film. In the study of the combination of pectin, konjac glucomannan and tea polyphenols, the incorporation of tea polyphenols notably increased the antioxidant ability of the film [30]. Ma et al. [52] found in a study on the preparation of slow-release antioxidant films using starch, potato peel polyphenols and chitosan nanoparticles that the addition of potato peel polyphenols improved the antioxidant performance of the film.

Free radicals are considered to be the main factor causing the oxidative deterioration of food. Therefore, packaging films capable of scavenging free radicals are of great significance in preventing food spoilage and extending its shelf life [53]. In this study, different concentrations of GSEE were added to the zein-based composite film, and the influence of this addition on the DPPH and ABTS free radical scavenging abilities of the composite film was investigated. The results (Figure 6b) show that, with the increase in the GSEE concentration, both the DPPH and ABTS free radical scavenging abilities of the composite film have been significantly improved. Specifically, when the GSEE concentration is 0 wt%, the DPPH and ABTS free radical scavenging rates are approximately 13 ± 0.72% and 8 ± 0.18%, respectively; while, when the GSEE concentration reaches 8 wt%, the DPPH and ABTS free radical scavenging rates increase by approximately 83.75% and 89.33%, respectively. This indicates that the flavonoids and proanthocyanidins in GSEE possess the function of free radical scavengers, which is in line with the research findings of the bioactive packaging system [54]. Likewise, other comparable studies have discovered that, in the experiment where the extract of Schinus terebinthifolia leaves was incorporated into the starch film, the scavenging activity of the film with the extract against DPPH and ABTS free radicals has been beefed up. When the concentration of the extract of Schinus terebinthifolia leaves increases to 15%, the scavenging rates of DPPH and ABTS free radicals increase by 88.52% and 90.16%, respectively. This improvement is positively associated with the rise in the extract concentration within the film [3].

### 3.8. Analysis of the Water Contact Angle (WCA)

**Figure 7:**
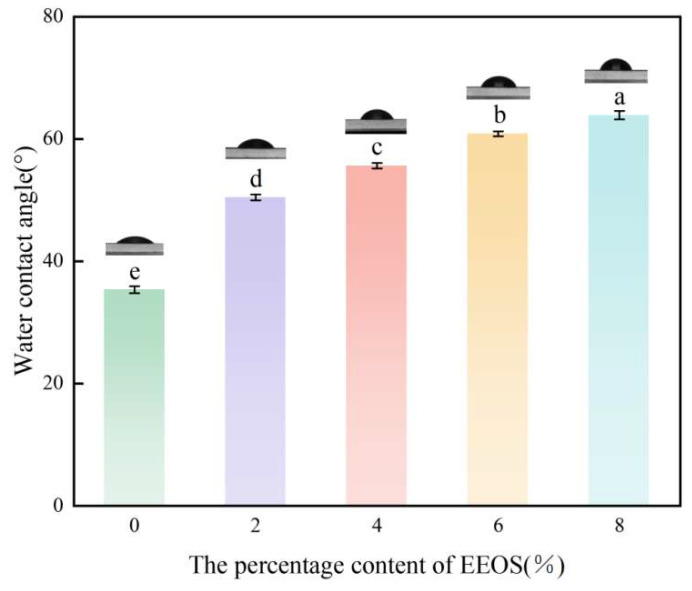
The WCA of Z/CA/GSEE−0% membrane, Z/CA/GSEE−2% membrane, Z/CA/GSEE−4% membrane, Z/CA/GSEE−6% membrane and Z/CA/GSEE−8% membrane. ^a–e^ Values are given as means + SD. Different letters in the figure indicate significant difference (*p* < 0.05).

The WCA on the polymer film’s surface serves as a crucial parameter for assessing its hydrophobic or hydrophilic characteristics. The greater the value of the water contact angle, the more pronounced the hydrophobicity of the film [7]. As depicted in Figure 7, with the increase in GSEE concentration, the WCA showed an increasing trend, reflecting the continuous enhancement of the film’s hydrophobicity. When the GSEE concentration reached 8 wt%, its WCA significantly increased by 44.65% compared with the control group without GSEE. This phenomenon was closely related to the microstructure of the film surface, as the aggregation of high-concentration GSEE further exacerbated the rough and uneven surface morphology, which was completely consistent with the observations from scanning electron microscopy (SEM) [55]. Similar results were found in a study on incorporating the natural antibacterial agent Glossy Privet Fruit Essential Oil (FEO) into zein films [7], where the addition of FEO enhanced the hydrophobicity of ZF films. While the addition of natural additives generally shows potential in modifying film properties, opposite results were observed in a study investigating the effects of alizarin/thymol on polycaprolactone/gelatin/zein nanofiber films, where the WCA value of the films decreased after adding thymol. This is because the phenolic hydroxyl groups in the thymol structure form strong hydrogen bonds with water molecules, making water droplets more likely to penetrate the film surface [56].

### 3.9. Antibacterial Activity

The development of antibacterial packaging materials is regarded as being of vital importance in prolonging the shelf-life of food. By applying antibacterial packaging technology, the original flavor and nutritional components of food can be maintained for a longer time, thereby minimizing the spoilage and waste resulting from microbial contamination [57]. Table 2 presents the antibacterial zone effects of zein-based composite films. These films were formulated using GSEE at various concentrations, ranging from 0 wt% to 8 wt%, against *Staphylococcus aureus* and *Escherichia coli.* When the concentration of GSEE is 0 wt%, the antibacterial zones for both *S. aureus* and *E. coli* are relatively small; as the concentration increases, a gradual increase in the antibacterial zones can be observed. This is likely because the GSEE contains various components with antibacterial activity, such as polyphenols like proanthocyanidins. These components may penetrate the lipopolysaccharide layer of the outer membrane, and citric acid enhances this effect by regulating the pH value and disrupting the extracellular polymers [41]. In addition, the diameter of the antibacterial zone of the Z/CA/GSEE film against *Staphylococcus aureus* was smaller than that against *Escherichia coli*, indicating that the GSEE exhibited a more significant inhibitory effect on Gram-negative bacteria (*Escherichia coli*) than on Gram-positive bacteria (*Staphylococcus aureus*). Liu et al. [58] prepared a composite antibacterial film (CAR film) using carvacrol and SPI as the primary raw materials. The results demonstrated that, with an increment in the amount of carvacrol added, the diameter of the antibacterial zone grew larger. In a study on preparing food packaging films by combining the bioactive extract of RRT with chitosan/zein, it was found that the coating film of the extract of RRT had good antibacterial activity [59].

### 3.10. Soil Degradation Test of the Composite Film

The degradation process of the film may be affected by environmental factors such as soil humidity, temperature, and the composition of the microbial community [60]. Table 3 presents the appearance changes in the zein-based composite film containing GSEE after 28 days of degradation in the soil. In the initial stage of degradation, that is, on the first day, the film shows a uniform light-yellow color, with a smooth surface and no apparent signs of degradation, indicating that the film’s structure is relatively stable at this time. When the degradation reaches the 7th day, some subtle texture changes begin to appear on the film’s surface, and the color becomes slightly darker, suggesting that the microorganisms and environmental factors in the soil have started to act on the film. By the 14th day, noticeable patchy changes appear on the film’s surface, and the color of some areas becomes darker, showing irregular erosion marks. When the degradation time reaches the 21st day, the degradation rate accelerates. By the 28th day, most of the areas of the film have presented a dark and fragmented state, and its structure has been severely damaged. In the research conducted by Medina Jaramillo et al. [61], a biodegradable and edible film of tapioca starch-glycerol was prepared, and the extract of Catharanthus roseus was added to the matrix as an antioxidant. With the passage of time, the film’s surface gradually changed from smooth to rough and fragmented, which is consistent with the degradation trend of the film in this study. This indicates that the addition of plant extracts may affect the interaction between the film and the microorganisms and other components in the soil environment, thus promoting the occurrence of the degradation process.

### 3.11. Measurement of the Peroxide Value (POV) in Lard

POV is one of the key parameters for evaluating the degree of oil oxidation and is often used to determine the freshness and stability of oils. The increase in the POV reflects the increase in the oxygen transmission rate [20].

**Figure 8:**
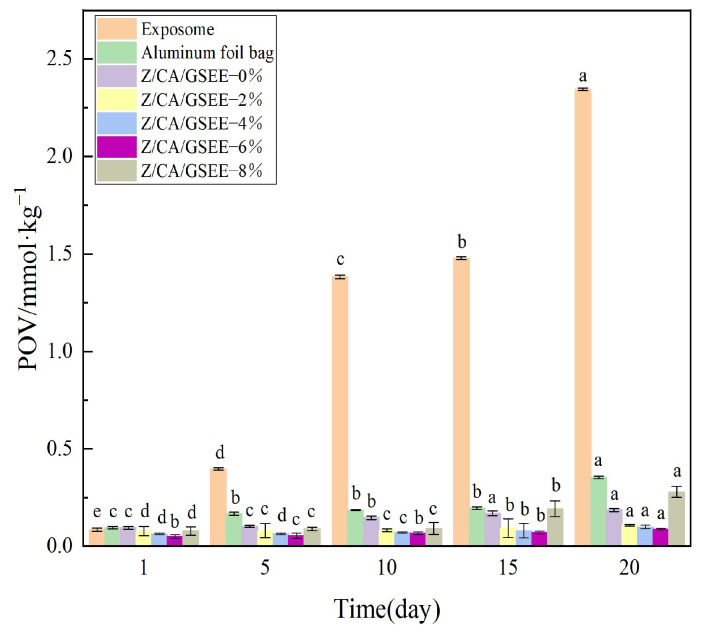
Peroxide value during the storage process in different packaging forms. ^a–e^ Values are given as means + SD. Different letters in the figure indicate significant difference (*p* < 0.05).

The changes in the POV of different treatment groups at various time points are shown in Figure 8. The control groups include the exposed group and the aluminum foil bag group, and the experimental group is the zein-based composite film group containing different concentrations of GSEE. As time progresses, the POV of all groups show an upward trend, which means that the oil gradually oxidizes during the storage process. At the same time point, the POV of the exposed group is markedly higher compared to those of other groups. This suggests that the rate of oil oxidation is rapid when the oil is left exposed without packaging. The POV of the aluminum foil bag group is significantly lower than that of the exposed group, which shows that the excellent barrier properties of the aluminum foil bag play a positive role in delaying oil oxidation. For the composite film group, when the GSEE concentration increases from 0 wt% to 6 wt%, the POV at each time point generally shows a downward trend, indicating that the Z/CA/GSEE-6% film has the best antioxidant effect. Nevertheless, during the latter part of the storage period (such as the 20th day), the POV of the Z/CA/GSEE-8% group is notably greater than that of other Z/CA/GSEE films. The results show that adding GSEE to the zein composite film can successfully inhibit oil oxidation. The GSEE is rich in antioxidant components such as polyphenols and flavonoids. These components can capture free radicals, interrupt the oxidation chain reaction, and thus reduce the POV [62]. This result is consistent with previous studies. Lv et al. [53] prepared a biodegradable active packaging film using sodium alginate and gelatin as the matrix and rose polyphenol extract (RPE) as the carrier. When the 0.3 RPE film was utilized for the packaging of edible oil, it substantially decreased the POV of the edible oil. Therefore, the Z/CA/GSEE composite film can be considered as a packaging material for foods with a high fat content, such as meat.

## 4. Conclusions

In the present research, bio-based composite films were fabricated using Z and CA as the base materials. GSEE was added at various concentrations ranging from 0 wt% to 8 wt%. Through a comprehensive evaluation of the films’ multiple properties, a series of valuable research results were obtained. When considering the physical and chemical properties, with the rise in the concentration of GSEE, there were significant increases in the thickness, WCA, and TPC of the composite films. The foregoing shows that adding GSEE effectively changed the physical and chemical nature of the composite film. Meanwhile, regarding antioxidant properties, it was fully demonstrated that GSEE significantly enhanced the antioxidant capacity of the composite films. The images obtained from SEM intuitively verified that, as the amount of GSEE increased, the surface and cross-sectional structures of the films gradually became rougher. When it comes to antibacterial characteristics, GSEE exhibited a more pronounced inhibitory effect on Gram-negative bacteria (*Escherichia coli*) compared to Gram-positive bacteria (*Staphylococcus aureus*), reflecting its potential broad-spectrum nature in antibacterial applications. Moreover, the lipid oxidation experiment demonstrated that the composite film with 6 wt% GSEE exerted a remarkable inhibitory impact on the oxidation of lard, which further supports the application potential of this composite film in food preservation. The results of the soil degradation experiment clearly showed that the composite film could achieve apparent degradation within a certain period in the natural environment, meeting the environmental protection requirements of biodegradable materials. In conclusion, the excellent performance of the Z/CA/GSEE composite film in terms of antioxidant properties, antibacterial properties, lipid oxidation inhibition ability, and soil degradability makes it show good application prospects in the field of biodegradable food packaging.

## Figures and Tables

**Table 1:** Thickness, color parameters, opacity, and images of composite films with different GSEE concentrations.

Concentration of the Extract	Thickness	L*	a*	b*	Opacity/%	Picture
0%	0.105 ± 0.02 ^e^	76.14 ± 0.24 ^a^	4.17 ± 0.19 ^d^	65.89 ± 0.33 ^a^	4.59 ± 0.02 ^e^	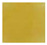
2%	0.109 ± 0.11 ^d^	74.60 ± 0.21 ^b^	4.53 ± 0.06 ^c^	40.49 ± 0.26 ^e^	5.01 ± 0.11 ^d^	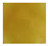
4%	0.120 ± 0.21 ^c^	73.59 ± 0.24 ^b^	4.68 ± 0.05 ^c^	44.66 ± 0.24 ^d^	6.45 ± 0.13 ^c^	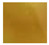
6%	0.135 ± 0.12 ^b^	70.64 ± 0.26 ^c^	7.62 ± 0.11 ^b^	46.72 ± 0.19 ^c^	7.54 ± 0.25 ^b^	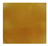
8%	0.152 ± 0.09 ^a^	60.25 ± 0.29 ^d^	17.51 ± 0.19 ^a^	49.79 ± 0.19 ^b^	8.09 ± 0.21 ^a^	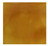

^a–e^ Values are given as means + SD. Different letters in the same line indicate significant difference (*p* < 0.05).

**Table 2:** Antimicrobial activities of the composite films against *S. aureus* and *E. coli*.

Composite Film	*Staphylococcus aureus* (mm)		*Escherichia coli* (mm)	
Z/CA/GSEE−0%	1.26 ± 0.12 ^e^	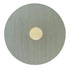	1.28 ± 0.04 ^e^	* 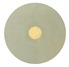 *
Z/CA/GSEE−2%	1.37 ± 0.28 ^d^	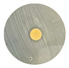	2.49 ± 0.17 ^d^	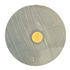
Z/CA/GSEE−4%	2.02 ± 0.33 ^c^	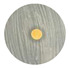	3.10 ± 0.09 ^c^	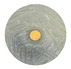
Z/CA/GSEE−6%	3.19 ± 0.12 ^b^	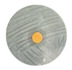	4.29 ± 0.31 ^b^	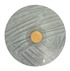
Z/CA/GSEE−8%	4.28 ± 0.19 ^a^	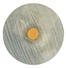	6.02 ± 0.22 ^a^	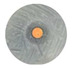

^a–e^ Values are given as means + SD. Different letters in the same line indicate significant difference (*p* < 0.05).

**Table 3:** Visual appearance changes in Z/CA/GSEE films after 28 days of degradation.

Degradation Time	1 Day	7 Day	14 Day	21 Day	28 Day
The change in the appearance of the film	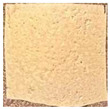	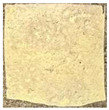	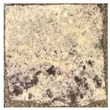	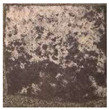	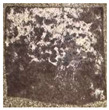

## Data Availability

The original contributions presented in the study are included in the article, further inquiries can be directed to the corresponding author.
